# The mammalian blastema: regeneration at our fingertips

**DOI:** 10.1002/reg2.36

**Published:** 2015-06-09

**Authors:** Jennifer Simkin, Mimi C. Sammarco, Lindsay A. Dawson, Paula P. Schanes, Ling Yu, Ken Muneoka

**Affiliations:** ^1^ Division of Developmental Biology, Department of Cell and Molecular BiologyTulane UniversityNew OrleansLouisiana70118USA

**Keywords:** Blastema, digit, hypoxia, mouse, regeneration, stem cells, wound healing

## Abstract

In the mouse, digit tip regeneration progresses through a series of discrete stages that include inflammation, histolysis, epidermal closure, blastema formation, and redifferentiation. Recent studies reveal how each regenerative stage influences subsequent stages to establish a blastema that directs the successful regeneration of a complex mammalian structure. The focus of this review is on early events of healing and how an amputation wound transitions into a functional blastema. The stepwise formation of a mammalian blastema is proposed to provide a model for how specific targeted treatments can enhance regenerative performance in humans.

## Introduction

Humans suffer organ damage and deterioration from aging, disease, and injury. The quest to reverse these processes has led to the identification of stem cells that can be used for cell replacement therapies, the development of a variety of different biomaterials that can be used to recruit cells in situ, and the combination of these two approaches. The stem cell/biomaterial approach represents the bulk of the current strategies targeting regenerative medicine (Carlson 2007; Place et al. 2009; Hench & Thompson 2010; Kirkpatrick 2014). The fact that there are clear successes in this field provides encouragement that human regeneration is an achievable goal. Since stem cells are derived from the adult body and decellularized adult organs can be used as a regenerative scaffold, the evidence is consistent with the conclusion that adult mammals, including humans, have the capability to mount a regenerative response (Macchiarini et al. 2008; Petersen et al. 2010). However, since mammals do not routinely regenerate, we can only point to a potential for regeneration that fails to activate in response to injury.

A key question is whether regenerative potential in mammals can be activated with the appropriate treatment or a series of treatments. In the past, to understand how mammals may regenerate we have turned to lower vertebrates such as fish, salamanders, frogs, and lizards that are known to be natural regenerators (Brockes & Kumar 2005; Gurtner et al. 2008). Fascination with the regenerative ability of these animals and the possibility of unleashing this power to humans graces the pages of comic books and science fiction movies, indicating that the prospect of human regeneration is at the forefront of our imagination. While mammals are not known for regenerative powers, a closer examination reveals the existence of a natural ability to regenerate that proceeds through processes similar to classic regeneration models. There are a handful of mammalian regeneration models and these include the digit tip of rodents (Borgens 1982; Han et al. 2008; Fernando et al. 2011), the closing of an ear hole punch in rabbits and some rodents (Joseph & Dyson 1966; Williams‐Boyce & Daniel 1980; Seifert et al. 2012), and the annual regrowth of deer antlers (Kierdorf & Kierdorf 2012). All three of these regeneration models traverse through a transient blastema stage in which undifferentiated cells accumulate and proliferate at the wound site prior to differentiating into the regenerated structure. These regenerative responses involve multiple tissue types that are organized into a functional replacement structure, and as such are governed by complex interactions between a variety of different cell types. Enhanced human regeneration is a significant goal for regenerative medicine and there is a growing interest in utilizing these mammalian regeneration models to dissect regulatory events that control regeneration. This may shed light on one of the great enduring mysteries of biology: why all animals create organs during development, but only some can recreate or regenerate organs as adults. One practical outcome of such studies is aimed at devising therapies to induce or otherwise enhance regenerative performance in humans. Successes in this area of research will revolutionize human medicine.

The focus of this review is on the mammalian blastema and we have used the mouse digit blastema as a regeneration model. Regeneration itself is a complex process that has a distinct temporal component and can be subdivided into discrete stages or phases (Simkin et al. 2013). It is generally accepted that developmental mechanisms govern the later stages of the regeneration response. For example, the differentiation of cell types during regeneration is not unlike cell differentiation during embryogenesis; thus late phases of regeneration are considered re‐development. There are caveats to the re‐development generalization, for example the digit tip of the mouse develops by endochondral ossification but regenerates by direct ossification (Han et al. 2008); however, the molecular basis of osteoblast differentiation itself is identical in both development and regeneration. At the same time, the early phases of regeneration involve the initial response to injury and parallel a wound healing response. In a very general sense, regeneration can be viewed as the successful transition between an adult wound healing response that interfaces with developmental processes. It is likely that such a transitional interface was selected for and evolved in animals that have regenerative powers. At this interface is the blastema: a transient developmental structure that arises from an adult wound healing response.

## Successful Regeneration is a Position‐Dependent Response

**Figure 1:**
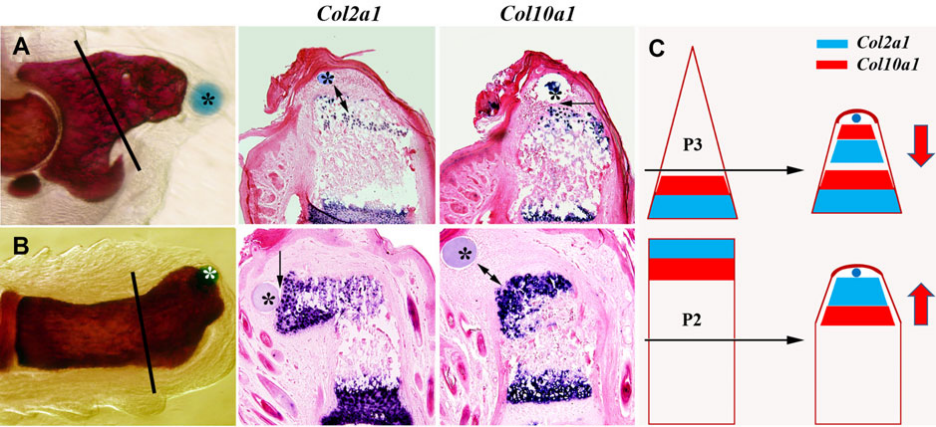
Position‐specific patterning is maintained in models of induced regeneration. (A) Proximal, non‐regenerating, P3 amputation is induced to regenerate by treatment with a microcarrier bead containing BMP2*. An endochondral ossification center forms during the regeneration response with proliferating chondrocytes expressing *Col2a1* proximal to hypertrophic chondrocytes expressing *Col10a1*. (B) BMP2 treatment of a mid‐level P2 amputation induces the regeneration of the distal half of the P2 element. The resulting regenerate displays an endochondral ossification center with proliferating chondrocytes expressing *Col2a1* distal to hypertrophic chondrocytes expressing *Col10a1*. (C) A schematic model showing the two regenerative responses displaying opposite polarity within the ossification centers induced by BMP2. Reprinted from Yu et al. (2012).

Most experimental models of regeneration involve the regrowth of a complex structure that initiates from an amputation injury and regenerates only those anatomical structures that were removed by amputation. Regeneration of different structures (e.g., arm or leg) or from different levels (e.g., forearm or digit) is necessarily distinct based simply on the structures that regenerate. Thus, one of the distinctions between the regenerative response and development is the specificity and variability of the response, and how the regenerated structure is able to smoothly interface with the amputated stump. The molecular underpinnings of this limb‐specific/level‐specific injury response remain an important but unsolved mystery that lies at the heart of the regeneration field (Bryant et al. 2002). Nevertheless, in the context of promoting mammalian regeneration, there is evidence that the mouse limb can be induced to regenerate in a level‐specific manner. Using a model of enhanced digit regeneration Yu et al. (2010) showed that Bone morphogenic protein 2 (BMP2) induces the regeneration of the third phalangeal element (P3) by endochondral ossification. In a subsequent study BMP2 similarly induced the regeneration of the second phalangeal element (P2) (Yu et al. 2012). In both cases skeletal regeneration was stimulated by inducing an endochondral ossification center at the amputation wound; however, the polarity of the two endochondral ossification centers was reversed and in accord with the development of these two digit structures (Fig. 1). Thus, BMP2 induces the regeneration of the P3 element by establishing an endochondral ossification center with proliferating chondrocytes proximal to hypertrophic chondrocytes, but induces regeneration of the distal P2 element by establishing an endochondral ossification center with proliferating chondrocytes distal to hypertrophic chondrocytes. Responses of opposite polarity induced by the same treatment provide clear evidence that cells of the amputation wound react to BMP2 in a distinct and position‐dependent manner. A similar conclusion based on anatomical responses can be drawn from enhanced regeneration following limb amputation (Ide 2012; Yu et al. 2012). Such studies show that the regenerative capability of mammals is not limited by the loss of a positional information system that was established during development. This conclusion is also supported by transcriptome analyses showing that adult human fibroblasts throughout the body maintain spatially distinct patterns of gene expression that other cell types lack (Chang et al. 2002; Rinn et al. 2006). Thus, current studies suggest that in the context of regeneration fibroblasts, present in the interstitial tissues of the body, function as a coordinate system to define spatial relationships between body parts and control the pattern of regenerative responses.

## The Regenerating Digit Tip as a Model for Blastema Formation in Mammals

**Figure 2:**
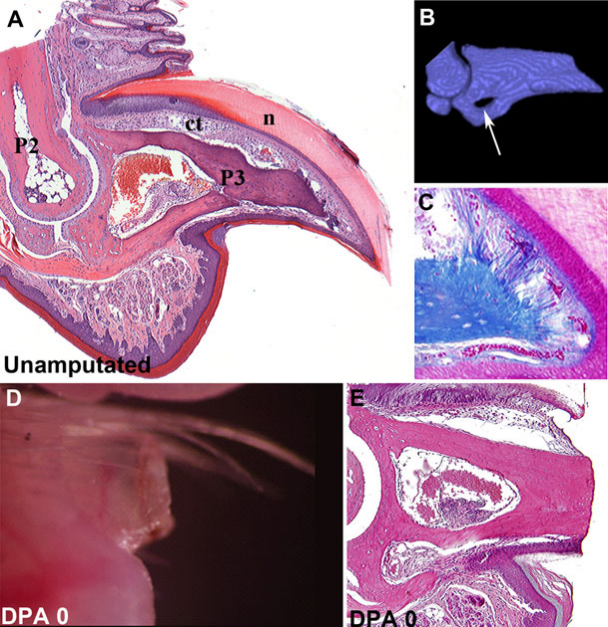
Architecture of the regenerative mouse terminal phalangeal element (P3) is unique. (A) Hematoxylin and eosin stain of the adult mouse P3 element shows its triangular shape with proximal bone marrow and articulation with the P2 element. The P3 bone is surrounded by loose connective tissue (ct) and the nail (n). (B) Three‐dimensional rendering of a μCT scan of the adult mouse P3 element showing the proximal os‐hole (arrow) and the tapered shape of the bone. (C) Mallory trichrome stains of the distal tip of the bone display fibrous connections between the tip of the P3 bone and the nail epidermis. (D) Brightfield image of the digit at the time of amputation. Amputation level is parallel to the fat pad and transects nail, epidermis, soft connective tissue, and bone. (E) Sections of the amputated digit tip showing the wound site. (D) is reprinted from Fernando et al. (2011) and (E) is reprinted from Simkin et al. (2013).

The digit tip of mice, primates, and humans is the only part of the mammalian limb that maintains regenerative capabilities at all developmental stages, including adulthood (McKim 1932; Douglas 1972; Illingworth 1974; Borgens 1982; Singer et al. 1987; Neufeld & Zhao 1995). The digit tip is defined by the P3 element, a bone that has a unique flattened conical shape with a basal bone marrow region and a pointed distal tip (Fig. 2A, B). The proximal end of the P3 element articulates with the distal end of the P2 element at the P2/P3 interphalangeal joint. The P3 element is partially encased within the nail organ, an epidermal structure that is required for the regenerative response (Takeo et al. 2013). The nail epidermis covers the dorsal and lateral aspect of the P3 element while the ventral epidermis is contiguous with the epidermis of the fat pad (Fig. 2A). Subjacent to the epidermal layer is the loose connective tissue that surrounds the P3 element. The proximal region of the P3 element has a unique foramen called the os‐hole that connects the marrow region to the proximal connective tissue (Fig. 2B). There is also a unique fibrous connection between the distal tip of the P3 element and the nail epidermis that is apparent with some connective tissue stains (Fig. 2C). The function of such connective tissue structures is unknown, but provides evidence of a structural link between the P3 bone and the overlying nail epidermis. Nerves and blood vessels are also present throughout the digit tip.

**Figure 3:**
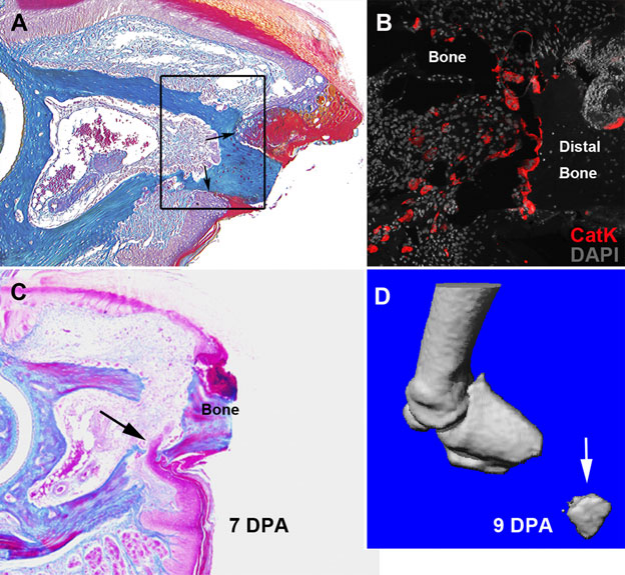
Histolysis and epidermal closure precedes blastema formation in the mouse digit tip. (A) One week after amputation in the adult mouse, the epidermal closure is prevented by the bone stump and the epidermis constricts around the amputated bone (arrow). (B) Multi‐nucleated osteoclasts expressing the degradation enzyme cathepsin K are prevalent both in the distal marrow and on the periosteal surface of the amputated bone. (C) Bone degradation and histolysis of surrounding tissues creates a path for epidermal migration (arrow) and opens the marrow cavity to the amputation wound. Epidermal closure causes re‐amputation and the release of the distal bone fragment. (D) Sloughing of the distal bone fragment (arrow) can be seen in μCT images between 1 and 2 weeks post‐amputation. (C) and (D) are reprinted from Fernando et al. (2011).

Amputation of the distal digit tip at a level even with the fat pad (Fig. 2D) causes minimal damage but initiates a highly reproducible sequence of events that leads to blastema formation and the replacement of the amputated tissues (Han et al. 2008; Fernando et al. 2011; Simkin et al. 2013). Amputation transects the P3 element without exposing the proximal marrow region so the amputation wound includes a centrally located bony region with a periphery of loose connective tissue encircled largely by the nail epidermis (Fig. 2E). While many regeneration models are associated with rapid epidermal closure, the wound epidermis of the amputated digit tip does not close rapidly or even over the amputated P3 element (Fernando et al. 2011). Instead, the epidermis constricts around the amputated P3 bone (Fig. 3A), there is a dramatic upregulation of osteoclasts that erode the distal bone (Fig. 3B), and the wound epidermis closes through the newly eroded bone (Fig. 3C). This results in a secondary amputation at a more proximal level and is associated with the release of the distal bone fragment that can be observed in histological and micro‐computed tomography (μCT) images of the regenerative response (Fig. 3C, D). Thus, epidermal closure occurs across a functional re‐amputation level that is significantly more proximal than the initial amputation. This injury induced re‐amputation erodes the distal bone and opens the bone marrow region to the amputation wound and a blastema forms soon after epidermal closure.

In amphibian limb regeneration the wound epidermis is known to be critically important for a successful regenerative response (Carlson 2007), and there is evidence that this is true for human fingertip regeneration (Illingworth 1974). The major epidermal structure of the digit tip is the nail organ, which is composed of the proximal nail matrix, the distal nail bed, and the overlying differentiated nail plate. Nail stem cells are localized in the nail matrix and give rise to proximal−distal columns of cells that extend into the nail bed and differentiate into the continuously elongating nail plate. The importance of the nail in regeneration is highlighted in a recent study showing that nail stem cell differentiation is Wnt‐dependent and that disrupting the canonical Wnt signaling pathway in the epidermis not only inhibits nail growth but also inhibits the skeletal regenerative response (Takeo et al. 2013). Additionally, gain‐of‐function studies provide evidence that activation of canonical Wnt signaling in the epidermis of proximal (non‐regenerating) P3 amputations induces nail and skeletal regeneration. Since the epidermis is well known to be essential for amphibian limb regeneration, the demonstration that mammalian regeneration is also dependent on the epidermis is perhaps not surprising, but does foster confidence that parallel strategies for regeneration have been maintained between evolutionarily diverse species.

The mouse digit tip blastema is characterized as a population of undifferentiated mesenchymal cells that form at the amputation injury site. Cells within the blastema proliferate at a rate that is significantly higher than the unamputated digit (Han et al. 2008; Fernando et al. 2011; Wu et al. 2013). Cells within the blastema express *Msx1*, a transcriptional repressor critical for differentiation, and genetic studies on fetal digit tip regeneration show that *Msx1* is required for regeneration (Han et al. 2003; Lehoczky et al. 2011). *Msx2* and *Dlx5* are also expressed in the regenerating fetal digit tip but regeneration is not impaired in the *Msx2* mutant, the *Dlx5* mutant, or the *Msx2*/*Dlx5* double mutant (Reginelli et al. 1995; Lee et al. 2013a). *Msx1* has been shown to be upstream of *Bmp4* expression in the digit tip, and experiments using the BMP antagonist Noggin show that BMP signaling is essential for both fetal and neonatal regeneration (Han et al. 2003; Yu et al. 2010). In proximal, non‐regenerative digit amputations, BMP treatment induces *Msx1* expression, enhances cell proliferation, and promotes a regenerative response (Han et al. 2003; Yu et al. 2010, 2012). These studies suggest that, like other regenerating model systems, repressing cell differentiation and inducing cell proliferation are critical for a successful mammalian regenerative response. Additional markers expressed by blastema cells and discussed in detail below include transcripts for pigment epithelium derived factor (*Pedf*), chemokine receptor type 4 (*Cxcr4*), and stromal cell derived factor 1 (*Sdf1*).

**Figure 4:**
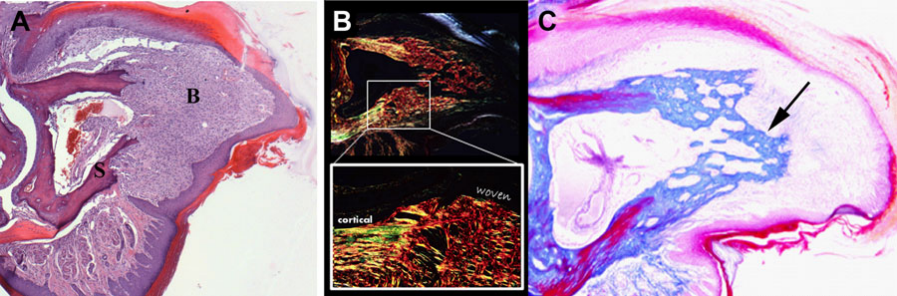
Blastema formation and redifferentiation. (A) The accumulation of a morphologically homogeneous population of cells at the distal edge of the degraded bone stump marks the blastema stage. The distinct transition between the differentiated bone of the stump (S) and the undifferentiated cells of the blastema (B) is evident in histological preparations. (B) Polarized light microscopy of picrosirius red stained sections of the redifferentiating digit tip reveals collagen fiber alignment in the original bone stump compared to the regenerate. The original bone shows parallel fiber alignment characteristic of cortical bone whereas new bone regenerates in a woven fashion with crosshatched collagen fibers. (C) The regenerated bone exhibits large trabecular spaces (arrow) which distinguish new bone from the original cortical bone stump. These trabecular spaces are continuously remodeled with time. (A) is reprinted from Simkin et al. (2015) and (C) is reprinted from Fernando et al. (2011).

The blastema that forms following adult digit tip amputation is composed of a morphologically homogeneous cell population condensed at the distal end of the amputated P3 bone (Fig. 4A). The marrow region of the P3 stump is filled with very large blood vessels and red blood cells appear to pool within the bone marrow. The vascularity of the blastema is unique in that the central region of the blastema just distal to the highly vascularized stump marrow appears avascular while blood vessels can be found in the blastema periphery. Cells expressing the endothelial marker CD31 are found in the avascular central region but these cells do not form vessels and co‐express stem cell antigen 1 (SCA1) suggesting that they are endothelial progenitor cells (Fernando et al. 2011). The abrupt transition from differentiated bone of the stump to the undifferentiated cells of the blastema is remarkable, and redifferentiation of the blastema initiates proximally building new bone directly onto the stump. Redifferentiation of the digit tip occurs by direct ossification and no chondrogenic cells are present during the regenerative response. The new replacement bone is termed woven bone and is characterized by numerous trabecular spaces that make it histologically distinct from the cortical bone of the stump (Fig. 4B, C). With time the regenerated woven bone increases in density and the trabecular spaces become smaller; however, the regenerate remains histologically distinct. Another unique characteristic of the regenerated digit tip is that the regenerated bone volume is consistently greater than the pre‐amputation bone volume (Fernando et al. 2011; Sammarco et al. 2014). Thus, the distinct woven bone and the consistent overshoot in bone volume indicate imperfections of this regeneration model and suggest the evolution of a functional outcome at the expense of an anatomically identical regenerate.

Despite the histological distinctions between regenerated and original bone, the return of P3 pattern is evident in the regenerate; for example the tapering morphology of the digit tip is restored (Fernando et al. 2011). Additionally, the dorsal curvature of the nail and positioning of the ventral fat pad is maintained in studies that involve amputation at more proximal levels (Rinkevich et al. 2011). While the regulation of dorsal−ventral patterning during digit regeneration has not been explored, there are a number of candidate genes known to regulate embryonic limb patterning that probably play a role. For example, engrailed‐1, a key regulator of dorsal−ventral limb patterning, is involved in directing proper innervation of the forelimb (Huettl et al. 2015) and is expressed during digit regeneration (Rinkevich et al. 2011). In addition, proper nerve regrowth plays a key role in the regenerative response (Takeo et al. 2013) and appears to be involved in patterning the regenerating bone (Rinkevich et al. 2014). It will be interesting to examine the control of dorsal−ventral patterning in digit regeneration in future studies.

One role that the wound epidermis plays in regulating the early events of blastema formation has been uncovered by experimentally altering the rate of wound closure (Simkin et al. 2015). The use of cyanoacrylates as a wound dressing in mammals improves the wound healing response in part by increasing the rate of epidermal closure (Singer & Thode 2004; Nipshagen et al. 2008; Singer et al. 2008; Wachter et al. 2010). Treatment of the amputated digit tip with Dermabond (2‐octyl cyanoacrylate) increases the rate of wound closure by about 50%. Thus, epidermal closure which normally occurs between 8 and 12 days post‐amputation (Fernando et al. 2011) is completed between 2 and 7 days in Dermabond‐treated amputation (Simkin et al. 2015). Regeneration still occurs following Dermabond treatment but the overall regenerative response is significantly altered. The wound epidermis closes directly over the amputated P3 bone surface, an outcome that is never observed following simple digit tip amputation (Fernando et al. 2011). During wound closure only the epidermis in Dermabond‐treated digits becomes hypoxic when assayed with Hypoxyprobe (2‐pimanidiazole‐HCl), dropping to an oxygen tension of less than 1.3%, and the Dermabond enhanced wound closure rate is reversed by subsequent treatment with hyperbaric oxygen (2.4 atmospheres absolute [ATA], 90 min, twice daily). These results show that oxygen availability and/or use influence epidermal cell behavior during a regeneration response and are consistent with previous studies in wound healing models (Winter 1963; Li et al. 2007; Mace et al. 2007; Botusan et al. 2008; Zimmermann et al. 2014). Secondary effects of Dermabond treatment include a reduced level of osteoclast maturation and a significant reduction in the level of post‐amputation bone erosion. The formation of a visibly smaller blastema is linked to this loss of degradation (Simkin et al. 2015). During wound healing and during development, tissue histolysis has multiple outcomes: the release of extracellular matrix fragments can act as mitogens or chemoattractants; the release of growth factors tethered to matrix molecules can promote cell division or differentiation; and the release of cells from the matrix can contribute to growth and repair (Daley et al. 2008). Dermabond studies suggest a thread linking epidermal wound closure to the regulation of the histolytic response and to the availability of either signaling factors and/or cells that can participate in blastema formation. The Dermabond studies also suggest that the size of the blastema scales with the amount of tissue that must regenerate to recreate missing structures.

## Cells of the Blastema

The cellular composition of the blastema is complicated by the early histolytic events that precede blastema formation. Cell lineage tracking studies show that a number of cell types participate in the regenerative response but remain lineage restricted (Lehoczky et al. 2011; Rinkevich et al. 2011; Wu et al. 2013). These cell types include epidermal cells which form a wound epidermis over the amputation site and give rise to epidermis‐derived cell types including the nail organ (Rinkevich et al. 2011; Takeo et al. 2013), endothelial cells which contribute to the regenerated vasculature (Lehoczky et al. 2011; Rinkevich et al. 2011), and osteoblasts which contribute to regenerated bone (Lehoczky et al. 2011). Because the post‐amputation osteoclast response creates an interface between the marrow space and the location of the forming blastema there is also the potential for the involvement of the bone marrow in regeneration. While many of the cell types within the bone marrow have yet to be tested, transplantation of labeled hematopoietic stem cells show that circulating cells do not contribute to major structural tissues of the regenerated digit (Rinkevich et al. 2011).

Additionally, the interstitial or fibroblastic cells of the loose connective tissues have not been carefully investigated and seem a likely source of a multipotent progenitor. Limb regeneration studies in which cellular contributions from other tissues are experimentally restricted provide evidence that cells of the connective tissue display a high level of plasticity (Dunis & Namenwirth 1977). In mammals, the perivascular fibroblasts (pericyte, mural cell, Rouget cell) represent a cell type involved in tissue repair (Schor et al. 1995; Doherty et al. 1998; Farrington‐Rock et al. 2004; Kalajzic et al. 2007; Xueyong et al. 2008). Perivascular fibroblasts function to support and provide rigidity to blood vessels, and are important for multiple steps of angiogenesis including endothelial cell proliferation, recruitment of smooth muscle cells, and stabilization of new vessels. The ability of perivascular fibroblasts to differentiate into a number of different cell types, including osteoblasts, chondroblasts, and adipocytes, is well documented in vitro. Because as a cell type the fibroblast is so poorly understood, sorting out its role in regeneration versus fibrosis (the scarring and wound healing response that is the antithesis of regeneration in mammals) represents a serious challenge for future studies.

**Figure 5:**
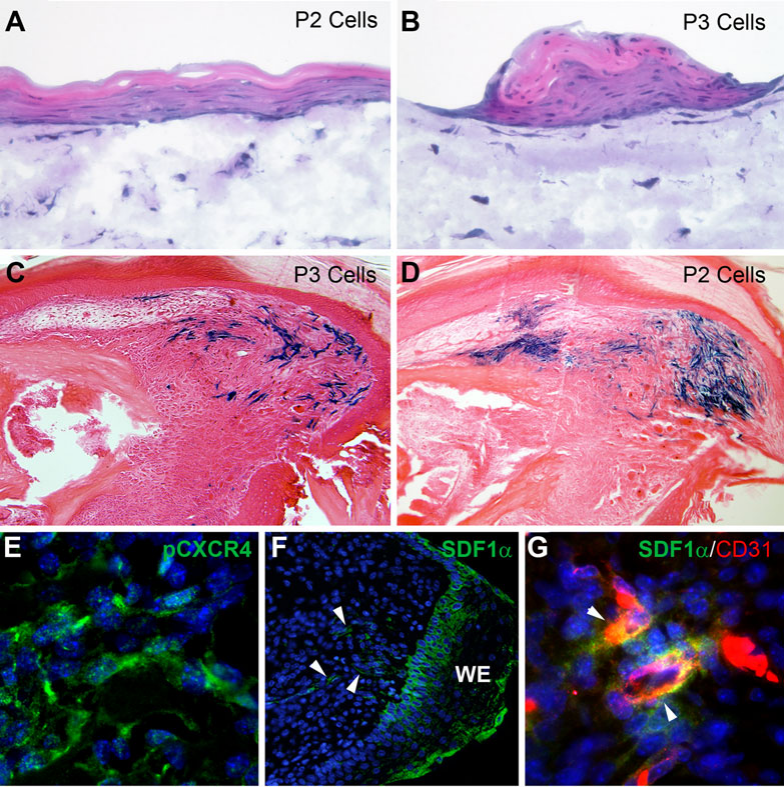
Migratory and position‐specific characteristics of blastema cells. (A) Experiments in which isolated P2 fibroblasts are co‐cultured with human keratinocytes show the induction of stratified sheets of differentiating epidermis. (B) Experiments in which isolated P3 fibroblasts are similarly co‐cultured with human keratinocytes show the induction of multilayered aggregates of keratinocytes that display nail‐like characteristics. (C) P3 cells derived from Rosa26R mice participate in blastema formation and digit tip regeneration when engrafted into the digit of SCID mice. (D) P2 cells derived from Rosa26R mice also participate in blastema formation and digit tip regeneration when engrafted into the digit of SCID mice. (E) Digit tip blastema cells immunostain positive for phospho‐CXCR4 (green) indicating that the SDF1α/CXCR4 signaling pathway is active during digit tip regeneration. (F) The CXCR4 ligand and chemoattractant, SDF1α (green), is expressed in the wound epidermis (WE) and in cells of the blastema (arrowheads) during the regeneration response. (G) SDF1α positive blastema cells (green) co‐express the endothelial marker CD31 (red) suggesting that endothelial cells of the blastema play a role in cell recruitment during digit tip regeneration. (A)−(D) are reprinted from Wu et al. (2013) and (E)−(G) are reprinted from Lee et al. (2013b).

To evaluate the role of fibroblasts in regeneration, Wu et al. (2013) isolated and characterized adult fibroblasts derived from the connective tissue of regeneration‐competent digit regions of the mouse and compared them to similar cells from regeneration‐incompetent regions. In this study, regeneration‐competent cells were derived from the P3 region and are called P3 cells, whereas regeneration‐incompetent cells were derived from the P2 region and are referred to as P2 cells. After isolation and expansion both P2 and P3 cells were maintained as cell lines and retained position‐specific characteristics. For example, in three‐dimensional organotypic skin equivalence co‐cultures with human keratinocytes, P2 cells induced the differentiation of a stratified epidermis similar to skin epidermis, whereas P3 cells induced keratinocyte aggregation into nail‐like structures (Fig. 5A, B). When re‐introduced back into the amputated P3 digit both P2 and P3 cells engraft and are able to participate in blastemal formation and the regenerative response (Fig. 5C, D), and both cell lines also participated in wound healing without regeneration after P2 amputation (Wu et al. 2013). These studies provide evidence that connective tissue fibroblasts possess and maintain stable position‐specific characteristics, and that these cells do not inherently dictate whether or not a regenerative response is induced at an amputation wound. Other in vitro studies using P2/P3 cell lines suggest unique differences in how these cells can modify the physical microenvironment of the blastema and, as well, initiate inductive signaling pathways known to be critical for a regenerative response (Lynch & Ahsan 2013, 2014).

In addition to enhanced cell proliferation, blastema cells characteristically express CXCR4, a receptor known to be associated with cell migration (Lee et al. 2013b). SDF1 (also called CXCL12) is a secreted chemoattractant and a member of the CXC chemokine family (Zlotnik & Yoshie 2000; Burns et al. 2006). There are two known G‐protein‐coupled SDF1 receptors, CXCR4 and CXCR7 (Burns et al. 2006), and both receptors are expressed by digit blastema cells (Fig. 5E) (Lee et al. 2013b). Many mammalian models of cell recruitment associated with an injury response implicate the SDF1/CXCR4 signaling pathway (Cantley 2005; Lama & Phan 2006; Ting et al. 2008; Bouzaffour et al. 2009). SDF1/CXCR4 signaling has also been implicated in blastema formation during fin regeneration in zebrafish (Bouzaffour et al. 2009). During mouse digit blastema formation SDF1 is expressed by the wound epidermis (Fig. 5F) and also by blastema cells that co‐express the endothelial cell marker CD31 (Fig. 5G). In vitro cell migration studies show that blastema cells are stimulated by SDF1 and this response is selectively inhibited by a CXCR4‐specific antagonist, AMD3100. Thus, while both CXCR4 and CXCR7 are expressed by blastema cells, it appears that SDF1 signaling via the CXCR4 receptor functions in the recruitment of cells to form the blastema. This conclusion is supported by gain‐of‐function studies in which the ectopic expression of SDF1 by cells engrafted into a non‐regenerating amputation wound stimulated the accumulation of CXCR4 positive cells and a partial regenerative response, and also by loss‐of‐function studies in which systemic treatment with AMD3100 inhibited the endogenous regenerative response (Lee et al. 2013b). Using a model in which regeneration from a non‐regenerative P2 amputation can be induced with BMP2 treatment (Yu et al. 2012), CXCR4 positive cell recruitment and SDF1 expression of CD31 positive endothelial cells was found to be induced by BMP2. Additionally, *Sdf1* transcripts were upregulated by BMP2 treatment of human microvascular endothelial cells (HMVEC) cells, and media conditioned by BMP2‐treated HMVEC cells stimulated blastema cell migration that was specifically inhibited by AMD3100. These studies provide strong evidence that cell recruitment is important for both endogenous and induced regenerative responses, and that SDF1/CXCR4 signaling plays a key role in this response.

## Hypoxia, Oxygen, and the Control of Regeneration

The relationship between the blastema and oxygen availability and use is apparent in both the regenerating axolotl limb model and the regenerating mouse digit model. Both axolotl (Peadon & Singer 1966; Mescher 1996) and mouse digit blastemas (Said et al. 2004; Fernando et al. 2011) have been shown to be avascular. More recently, the mouse digit blastema was shown to be specifically hypoxic, an integral event that is part of a dynamic changing oxygen environment during digit regeneration (Sammarco et al. 2014). Curiously, the bone degradation phase that precedes the blastema phase shows a hypoxic microenvironment associated with the marrow bone lining cells, and the bone regeneration phase that follows the blastema phase shows hypoxic microenvironments only in the trabeculae of newly forming bone (Sammarco et al. 2014). Disruption of this hypoxic event with the application of hyperbaric oxygen exacerbates the degradation phase and delays the transition from blastema to bone. This suggests that relief from a hypoxic environment is just as critical for successful regeneration as the hypoxic blastema environment itself.

HIF‐1 (hypoxia inducible factor) is the primary intermediary in cell survival and metabolism during hypoxia (Semenza 2003). The hypoxic environment of the blastema is consistent with the findings that both SDF1 (Ceradini et al. 2004) and CXCR4 (Staller et al. 2003; Speth et al. 2014) are upregulated by HIF in hypoxic conditions. Studies on human umbilical vein endothelial cells (HUVECs) have shown that hypoxic conditions increase the number of adherent endothelial progenitor cells (EPC) in an SDF1/CXCR4‐dependent manner and pattern (Ceradini et al. 2004). Interestingly, in in vivo studies where CXCR4 positive EPCs were engrafted into nude mice after ischemic surgery, there was no significant engraftment if the EPCs were administered after tissue oxygen tension had been restored (Ceradini et al. 2004). Thus, it is likely that the integral hypoxic microenvironment during the blastema phase serves as a basis for hypoxic cellular trafficking cascades, including SDF1/CXCR4 signaling, which in turn serve to enhance cell recruitment and retention, and facilitate neovascularization.

While a hypoxic event is necessary for blastema formation, release into a normoxic environment with adequate oxygen levels is just as critical. Fourteen days post‐amputation shows hypoxic areas restricted to the trabeculae of newly forming bone and ex vivo digit slice culture also shows that increased oxygen is conducive to the mineralization of bone (Sammarco et al. 2014). Threshold oxygen levels are required for the hydroxylation (Fessler & Fessler 1974; Utting et al. 2006) and subsequent secretion (Ramaley & Rosenbloom 1971) of collagen from osteoblasts in order to generate mineralized bone matrix. Thus, the changing oxygen microenvironment during regeneration is likely to give clues as to the molecular mechanisms operating downstream from this primary cue. Furthermore, utilizing oxygen as a manipulative force may also aid in moving the field forward with regard to controlling the stepwise pattern of regeneration.

## Angiogenesis and the Digit Blastema

The hypoxic blastema phase is probably generated, at least in part, by the fact that the blastema is largely void of blood vessels. Since there is evidence that endothelial cells of the stump vasculature give rise to endothelial cells of the regenerate (Lehoczky et al. 2011), and that most of these individual blastemal endothelial cells also express SCA1 (Fernando et al. 2011), it is likely that endothelial progenitor cells associated with injured vessels of the stump enter the blastema but are transiently inhibited from reforming new vessels. This is consistent with the finding that transcripts of the pro‐angiogenic growth factor *Vegfa* are downregulated in the blastema. However, the attenuation of *Vegfa* expression runs counter to the hypoxic nature of the blastema and the well‐characterized HIF‐1 regulation of *Vegfa* expression. Concurrent with the finding that the blastema is void of *Vegfa* expression is the discovery that many blastema cells express transcripts for the anti‐angiogenic factor *Pedf* (Muneoka et al. 2008). The expression of *Pedf* and the lack of expression of *Vegfa* within the blastema are consistent with other studies demonstrating the anti‐angiogenic properties of pigment epithelium derived factor (PEDF) (Cai et al. 2011; Liu et al. 2012).

While non‐regenerative digit amputation wounds display a much reduced level of *Pedf* transcripts, induced regeneration models where BMP2 and BMP7 are applied to the regeneration‐incompetent P2 amputation site at the time of wound closure demonstrate increased levels of *Pedf* transcripts (Yu et al. 2010, 2012). Additionally, in studies where vascular endothelial growth factor (VEGF) is applied to the regenerating digit tip, regeneration is completely inhibited suggesting that precocious angiogenesis creates a regeneration‐incompetent wound environment (Yu et al. 2014). Collectively, these data suggest that the early expression of PEDF may be functioning to temper certain downstream activities independent of canonical hypoxic signaling. It is interesting to note that VEGF inhibition of regeneration does not inhibit blastema formation itself, but seems to inhibit the subsequent stage of redifferentiation. This suggests that the avascular microenvironment of the regenerating wound is not required for the blastema itself, but is required for the transition of the blastema to the differentiative phase of regeneration. The data suggest that avascularity within the blastema is required to create a microenvironment that is conducive for the intercellular signaling required to complete the regeneration response (Yu 2014).

## Inflammation and Blastema Formation

The main components of the amputation microenvironment leading to blastema formation are inflammatory cells. In wound healing models, the macrophage, the main cellular orchestrator of the inflammatory environment, performs essential roles to promote healing. The wide range of macrophage functions during wound healing include the secretion of proteases like matrix metalloproteinases to degrade matrix and clear the tissue of debris, the phagocytosis of apoptotic cells, the release of chemoattractants to promote cell migration and angiogenesis, and the secretion of growth factors to induce cell division, differentiation, and/or matrix production (Rappolee et al. 1988; Willenborg et al. 2012; Novak & Koh 2013; Wynn et al. 2013). When macrophages are depleted in mouse dorsal wound healing models, epidermal closure is inhibited and the wound is marked by a decrease in myofibroblasts and keratinocyte migration (Leibovich & Ross 1975; Lucas et al. 2010). Without macrophages, apoptotic cells dominate the wound tissue and wounds exhibit a rise in reactive oxygen species like hydrogen peroxide. Additionally new blood vessel growth is significantly decreased as are levels of angiogenic factors like VEGF and transforming growth factor β (DiPietro 1995; Khanna et al. 2010; Koh & DiPietro 2011; Murray & Wynn 2011; He & Marneros 2013).

While macrophages have overlapping roles with other cells in the wound environment, emerging evidence suggests the specific source of growth factors contributes to differences in wound healing abilities. VEGF expression, for example, is dominated by macrophages in early stages of dorsal skin wounds and in later stages by the wound epidermis. A monocytic source of VEGF promotes leaky vessels whereas an epidermal source provides a more comprehensive network of vessels covered with pericytes (Rossiter et al. 2004; Willenborg et al. 2012). These processes are essential not only to wound healing but also to events leading up to blastema formation. Speculating that macrophages in general are either inhibitory or essential for regeneration is too broad a statement because so many functional phenotypes of macrophages exist (Mosser 2003; Murray & Wynn 2011; King et al. 2012; Novak & Koh 2013). However, complete depletion of macrophages from wound healing and regenerating systems tends to have more of a negative impact than positive (Alexander et al. 2011; Xiang et al. 2012; Li et al. 2013). Complete depletion of macrophages from early wounds inhibits limb regeneration in the axolotl and heart regeneration in neonatal mice (Godwin et al. 2013; Aurora et al. 2014). It is therefore likely that a subtype of macrophages will play a large role in orchestrating blastema formation in mammalian regenerating models as well.

## Summary: How to Make a Mammalian Blastema

**Figure 6:**
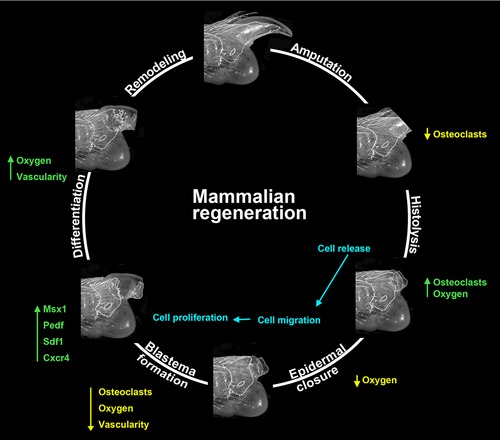
How to make a mammalian blastema. Histolysis of bone and surrounding tissue set up cellular availability for blastema formation and release factors into the microenvironment. Cell migration and proliferation contribute to the accumulation of progenitor cells which form the blastema. Blastema formation is framed in part by fluctuations in osteoclast activity and oxygen tensions of the tissue. The blastema itself exists in a hypoxic, avascular microenvironment and is characterized by the re‐expression of developmental genes like *Msx1* and the expression of the anti‐angiogenic factors like PEDF. The proper control of timing and the successful completion of each stage leading up to blastema formation are critical for regenerative ability.

In considering how to make a mammalian blastema it is clear that, immediately following amputation injury, cells of the wound site must transition through multiple sequential phases (Fig. 6). The blastema is a cellular aggregate of multiple cell types that become organized into the various tissues of the regenerate. In principle, a minimum of three events must be regulated to successfully build a blastema. First, there must be cells available that can participate in blastema formation. Blastema cells can come from a variety of different tissues and tissue‐specific progenitor cells represent an obvious cell source. We propose that the histolytic response during the early phases after amputation plays a key role in degrading tissues and increasing the availability of progenitor cells. During this phase high oxygen levels and the absence of the inhibitory influence of the wound epidermis extends the histolytic process. In the case of fetal or neonatal regeneration, we suggest that the availability of progenitor cells is not a limiting factor because the digit is still developing. This accounts for the observed enhanced regenerative response among children, and also for successes in induced regenerative responses by BMPs (Yu et al. 2010; Ide 2012; Yu et al. 2012). Histolytic activity also plays a key role in modifying the existing tissue substrate in preparation for integrating newly regenerated tissues.

Second, progenitor cells must be recruited to the amputation wound site where they can aggregate to form the blastema. Cell labeling studies are consistent with significant cell migration during blastema formation (Wu et al. 2013). The SDF1/CXCR4 signaling pathway plays a key role in recruiting cells during a regenerative response and is therefore a prime candidate for enhancing cell recruitment following injury (Lee et al. 2013b). SDF1 is expressed by both the wound epidermis and endothelial cells of the blastema, and blastema cells express CXCR4 and migrate in response to SDF1. Known inducers of the regenerative response (i.e., BMP2) upregulate SDF1 expression that subsequently recruits CXCR4 positive cells to the amputation wound. Third, proliferation of progenitor cells within the blastema must be stimulated to increase blastema size prior to differentiation. Treatment with BMP7 or BMP2 stimulates blastema cell proliferation and can induce regenerative responses (Yu et al. 2010, 2012). Wnt signaling during the regenerative response is also linked to enhanced proliferation (Takeo et al. 2013).

An additional aspect important for a regenerative response is the control of the microenvironment within the blastema. The amputated digit stump progresses from high oxygen tension prior to blastema formation to a hypoxic environment within the blastema (Sammarco et al. 2014). We propose that oxygen tension plays a key role in regulating phase transitions important for the regenerative response; early on oxygen regulates the transition from histolysis to blastema formation, and later from blastema to differentiation. One way that oxygen is regulated is by modulating neovascularization as the blastema forms. Unlike other wound healing models (Wietecha et al. 2013), *Vegfa* expression is not upregulated early in regeneration and treatment with VEGF enhances neovascularization and inhibits the regenerative response (Yu 2014). One unique feature of the digit blastema is that many cells express the anti‐angiogenic factor PEDF. PEDF treatment inhibits *Vegfa* expression by blastema cells and rescues regenerative failure resulting from induced *Vegfa* expression. The data are consistent with a model in which regulating angiogenesis modulates oxygen availability and changes in oxygen tension signal phase transitions critical for the regenerative response.

In the context of regenerative medicine, it is important to recognize that failure to transition from any one phase to the next would result in regenerative failure, so a “one treatment fits all” outcome seems unlikely. In other words, a single “magic bullet” for human regeneration is probably not a reasonable goal, but treatments involving sequential interventions that navigate the injury/healing/repair response are likely to result in therapies to enhance endogenous regenerative capabilities. In addition, it is also clear that mechanisms associated with the mammalian regenerative response involve the regulation of pathways that are intricately linked to a large number of human disease states (e.g., osteoporosis, fibrotic tissue diseases, cancer); thus an understanding of how the body regulates these pathways in a positive way will inform future therapeutic approaches.
